# Diagnosis of the risk of occupational disease among outsourced workers

**DOI:** 10.47626/1679-4435-2023-1092

**Published:** 2024-09-24

**Authors:** Leonardo Faria Martins, Ana Carolina Mesquita do Nascimento, Sergio Valverde Marques dos Santos, Luiz Almeida da Silva

**Affiliations:** 1 Instituto de Geografia, Universidade Federal de Uberlândia, Uberlândia, MG, Brazil; 2 Programa de Pós-Graduação em Gestão Organizacional, Universidade Federal de Catalão (UFCAT), Catalão, GO, Brazil; 3 Escola de Enfermagem de Ribeirão Preto, Universidade de São Paulo, Ribeirão Preto, SP, Brazil; 4 Programa de Pós-Graduação em Gestão Organizacional, Departamento de Enfermagem, Instituto de Biotecnologia, UFCAT, Catalão, GO, Brazil

**Keywords:** occupational health, work conditions, outsourced services, occupational risks, saúde do trabalhador, condições de trabalho, serviços terceirizados, riscos ocupacionais

## Abstract

**Introduction:**

The outsourcing of work activities has caused new and precarious working conditions, impacting the health and safety of workers, resulting in an increase in disease and accidents given the vulnerability established in the contemporary labor market.

**Objectives:**

To identify the occurrence of risk of disease resulting from work, among outsourced workers.

**Methods:**

Quantitative, observational, analytical and cross-sectional study, with application of the Inventário de Trabalho e Riscos de Adoecimento in 187 workers of a company that supplies and manages human resources for third parties, under contract with a Federal University, located in the state of Minas Gerais.

**Results:**

The Inventário de Trabalho e Riscos de Adoecimento obtained the following averages for the following factors: work organization, 2.9 (standard deviation = 0.6) (critical); working conditions, 2.4 (standard deviation = 0.7) (critical); physical cost, 3.9 (standard deviation = 0.6) (severe); physical damage, 2.1 (standard deviation = 1.3) (critical).

**Conclusions:**

The participating workers showed a good perception associated with the Inventory of Inventário de Trabalho e Riscos de Adoecimento factors, resulting in diagnoses with the presence of risks of disease and accidents resulting from work.

## Abstract (pt)

**Introdução:**

A terceirização das atividades laborais tem provocado novas e precárias condições de trabalho, impactando na saúde e segurança dos trabalhadores e acarretando o aumento de adoecimentos e de acidentes, dada a vulnerabilidade estabelecida no mercado de trabalho contemporâneo.

**Objetivos:**

Identificar risco de adoecimento decorrente do trabalho entre trabalhadores terceirizados.

**Métodos:**

Estudo quantitativo, observacional, analítico, transversal com aplicação do Inventário de Trabalho e Riscos de Adoecimento a 187 trabalhadores de uma empresa de fornecimento e gestão de recursos humanos para terceiros sob contrato com uma universidade federal localizada no estado de Minas Gerais.

**Resultados:**

O Inventário de Trabalho e Riscos de Adoecimento obteve as seguintes médias para os fatores a seguir: organização do trabalho, 2,9 (desvio-padrão = 0,6) (crítico); condições de trabalho, 2,4 (desvio-padrão = 0,7) (crítico); custo físico, 3,9 (desvio-padrão = 0,6) (grave); danos físicos, 2,1 (desvio-padrão = 1,3) (crítico).

**Conclusões:**

Os participantes apresentaram boa percepção associada aos fatores do Inventário de Trabalho e Riscos de Adoecimento, resultando em diagnósticos que apresentaram risco de adoecimentos e de acidentes decorrentes do trabalho.

## INTRODUCTION

Global economic changes since 1970 have had repercussions on the working class to this day, as they have shaped workers’ profiles, contributing to fewer permanent jobs – the hallmark of the industrial working class – and a growing number of more flexible jobs.^1^

New (and poor) working conditions, new technologies, and new employment relationships (characterized by flexible working times and schedules) have led to the emergence and increase of new occupational diseases and accidents. The low costs involved in outsourcing have led investors to focus on what will yield a profit, reducing resources for health preservation, and occupational safety and health (OSH). Thus, in many cases, outsourced workers are more vulnerable to environmental risks than their counterparts.^2^

In Brazil, OSH regulations cover physical, chemical, biological, ergonomic, and mechanical risks, with the sole purpose of preventing accidents and diseases that cause, for example, bodily harm, chemical poisoning, microbiological contamination, musculoskeletal wear and tear, among other things that are within the worker’s body. These standards end up excluding work-related accidents and psychological disorders.^3,4^

Poor environmental working conditions in outsourcing activities are commonly and generally pointed out in various studies as one of the causes of the increased risk of accidents and diseases. However, previous research has shown no correlation and in-depth identification of specific OSH issues that are not aligned with existing prevention standards.^5,6,7,8,9,10^

Given the above and the limited number of studies that have investigated the subject of outsourced workers and the risks of becoming ill, this study is important as it aims to provide scientific information on this population in the search for better living and working conditions. Therefore, this study aimed to identify the risk of work-related diseases among outsourced workers.

## METHODS

This is a quantitative, observational, analytical and cross-sectional study including workers at a company that supplies and manages human resources for third parties on a contract with a federal university located in Minas Gerais, Brazil. The contract covers the provision of janitorial and maintenance services on the four campuses.

The population consisted of outsourced workers in janitorial and building conservation roles, working as general assistants, foremen, and glass cleaners, totaling a population of213 workers. A population study was chosen to include all workers who met the inclusion criteria, reaching a sample of 187 employees.

The inclusion criterion to choose participants in the study was employees who were permanently based at the campuses, regardless of their role/position. Employees who did not work permanently at the campuses, for example, who were replacing employees on vacation or on leave for various reasons, were excluded. In addition, when the questionnaires were completed, participants who self-reported as completely illiterate and who had been employed for less than 30 days were also excluded.

This study used a validated data collection tool, the Inventário de Trabalho e Riscos de Adoecimento (ITRA, Inventory of Work and Disease Risks), which was developed by Ferreira & Mendes^11^ to help diagnose critical indicators at work. After re-evaluations and improvements, the inventory was updated to its latest version^12^ in 2007, using factor analysis, the principal axis factoring (PAF) method, oblimin rotation, and factor reliability analysis using Cronbach’s alpha. According to the authors, it is possible to “[...] investigate work and associated risks of disease in terms of representation of the work setting, demands (physical, cognitive, and affective), experiences and damage” (Mendes & Ferreira, p. 112).^12^

Mendes & Ferreira^12^ suggest that the results can be used for the following purposes: “In research applications, it will be useful for those willing to investigate large populations and organizations, but also for researchers aiming to develop diagnostic health research, with a view to implementing prevention, occupational health, and Quality of Life at Work (QOLW) programs.”

The following factors from the scales were used to verify the risks of disease in line with occupational health, as described below.

### ESCALA DE AVALIAÇÃO DO CONTEXTO DE TRABALHO (EACT, WORK SETTING ASSESSMENT SCALE) - FACTORS: WORK ORGANIZATION AND WORKING CONDITIONS

The first factor has 11 affirmative items and seeks to measure the strictness/intensity of how work is conducted, while the second factor has 10 affirmative items and seeks to assess the physical structure and material conditions to which workers are subjected. These two EACT factors are measured using a 5-point Likert scale, ranging from “never” to “always.” This scale and all factors have eigenvalues of 1.5, total variance of 38.46%, Kaiser-Meyer-Olkin (KMO) of 0.93 and factor loadings above 0.30. Cronbach’s alphas were 0.72 for work organization and 0.89 for working conditions. The items in each factor were evaluated using a 5-point frequency scale, with negative items, whose factor score is obtained through the mean between the items. It is to be analyzed using three levels that include the midpoint and standard deviations (SD) in relation to the midpoint. This classification includes severe (factor score above 3.70), moderate or critical (scores between 2.30 and 3.69), and positive or satisfactory (score below 2.30) levels.^12^

### HUMAN COST AT WORK SCALE (ECHT) - FACTOR: PHYSICAL COST

This factor has 10 affirmative items that measure the demand for activities requiring workers to move physically, to indicate physiological and biomechanical wear and tear during the working day. The ECHT factor is also measured using a 5-point Likert scale, from “never” to “always.”^12^ This scale and its factor analysis have eigenvalues above 2.0, total variance of 44.46%, KMO of 0.91, and factor loadings above 0.30. The Cronbach’s alpha is 0.91. The items in this factor were assessed using a 5-point human cost of work indicator demand level scale, with negative items, and the factor score is obtained through the mean of the items. It is to be analyzed using three levels that consider the midpoint and the SD in relation to the midpoint. This classification involves severe (factor score above 3.70), moderate or critical (scores between 2.30 and 3.69), and positive or satisfactory (score below 2.30) levels.^12^

### WORK-RELATED HARM ASSESSMENT SCALE (EADRT) - FACTOR: PHYSICAL HARM

This factor has 12 affirmative items covering work-related pain and biological disorders. This EADRT factor is measured using a 7-point Likert scale, from “not at all” to “six times or more,” considering the last three working months at the time of the survey. This scale and its analysis factor have eigenvalues of 1.5, total variance of 50.09%, KMO of 0.95, and factor loadings above 0.30. The Cronbach’s alpha is 0.8812.

A four-level analysis was performed, considering the midpoint in a split into two intervals, applying an SD range. For harm indicators, this classification involves the most negative assessment levels (factor score above 4.10), the most serious (between 3.10 and 4.0), moderate or critical (between 2.0 and 3.0), and the most positive or acceptable assessment (score below 1.99) levels.^12^

This scale sought to specifically assess the occurrence (once, twice, or more) of indicators of work-related harm in the last three working months. Accordingly, the questionnaire used three scales, divided into four factors, totaling 48 affirmative items assessed.

The employees of the contractor completed the questionnaire independently at their workplace in May and June 2019. The investigator was not directly involved in the completion of the questionnaire, and was only present to provide guidance and clarify any questions about the survey.

All the meetings to complete the questionnaire were previously booked, according to time and personnel availability, so that groups of three or more workers who voluntarily agreed to participate could complete their questionnaires. This was done so as not to disrupt the contractee’s activities.

We analyzed the data using Stata version 14.0. Initially, the Kolmogorov-Smirnov test with Lilliefors correction ensured the normality of the study’s quantitative variables. A descriptive analysis of the outsourced professionals’ work variables was then performed. The study’s quantitative variables were presented as mean and SD, median and interquartile range (IQR) in the absence of normality.

The internal consistency of the ITRA subscales was analyzed using two tests. To analyze internal consistency, the standardized Cronbach’s alpha coefficient was used for each evaluation period. Values > 0.7 suggest good internal consistency.^13^ The intraclass correlation coefficient (ICC) was also used to analyze consistency. Values below 0.5, between 0.5 and 0.75, between 0.75 and 0.90, and > 0.90 indicate poor, moderate, good, and excellent reliability, respectively.^14^ A confidence level of 5% (p ≤ 0.05) was set for all analyses.

In accordance with Resolution No. 466 of December 12, 2012 of the Conselho Nacional de Saúde (CNS, Brazil National Health Council), which regulates research with human beings, the project for this study was submitted to the Comitê de Ética em Pesquisa (CEP, Research Ethics Committee) of the Universidade Federal de Uberlândia, and data collection began only after approval of the project under opinion No. 3.289.573.

## RESULTS

Table 1 shows the sociodemographic and work profile of the group of workers investigated.

**Table 1:** Sociodemographic and work profile of outsourced professionals. Uberlândia, MG, Brazil, 2019

Variables	n = 187	%
Qualitative		
Sex		
Female	172	92.0
Male	15	8.0
Marital status		
Single	56	29.9
Married/consensual union	109	58.3
Separated/divorced/widowed	22	11.8
Educational background		
Less than elementary school*	95	50.8
Elementary school/less than high school	45	24.1
High school or more	47	25.1
Role		
General assistant	170	90.9
Foreman	8	4.3
Glass cleaner	9	4.8
Other income		
Yes	27	14.4
No	160	85.6
Quantitative	Mean (SD)	Median (IQR)
Age (years)	40.9 (10.1)	42.0 (33.0-47.0)
Income (BRL)	1,058.4 (148.6)	1,000.0 (960.0-1.133.0)
Length of service (years)	3.1 (2.7)	2.4 (1.1-4.5)

SD = standard deviation; IQR = interquartile range; BRL = Brazilian Reais.

*Illiterate and less than elementary school.

According to Table 2, the main findings of the intended characteristics were predominantly women (92%), married persons (58.3%), and less than elementary school (50.8%). The mean age of the participants was 40.9 years (SD: 10.1) and the median was 42.0 (IQR: 33.0-47.0). As for their role, most were general assistants (90.9%); the mean and median length of service was 3.1 years (SD: 2.7) and 2.4 years (IQR: 1.1-4.5), respectively. The mean income was BRL 1,058.4 (SD: 148.6) and the median was BRL 1,000.00 (IQR: 960.0-1,133.0); only 14.4% reported having an additional income.

**Table 2:** Analysis of ITRA factors in outsourced professionals, Uberlândia, MG, Brazil, 2019

Factors	Mean (SD)	Score	95%CI	Median	IQR	Value		Cronbach’s alpha	ICC	p-value*
						Low	High			
Work organization	2.9 (0.6)	Critical	2.8-3.0	2.9	2.5-3.4	1.6-4.6		0.660	0.655	< 0.001
Working conditions	2.4 (0.7)	Critical	2.3-2.5	2.4	1.9-2.9	1.0-4.8		0.727	0.723	< 0.001
Physical cost	3.9 (0.6)	Severe	3.8-4.0	4.1	3.6-4.4	2.3-5.0		0.742	0.733	< 0.001
Physical harm	2.1 (1.3)	Critical	1.9-2.3	2.1	1.0-3.0	0.0-5.0		0.839	0.846	< 0.001

ICC = intraclass correlation coefficient; SD = standard deviation; IQR = interquartile range; ITRA = Inventário de Trabalho e Riscos de Adoecimento (Inventory of Work and Disease Risks).

*F-test.

Table 2 shows the descriptive analysis and internal reliability of the four factors of the ITRA three sub-scales, showing the main results, which are fundamental to determine the work-related risk of disease. The F-test for all the factors in Table 2 resulted in the data being statistically significant.

The work organization and working conditions factors at EACT had means of 2.9 (SD: 0.6) and 2.4 (SD: 0.7) respectively, a critical risk of disease. The EADRT physical harm factor had a mean of 2.1 (SD: 1.3), a critical risk of disease (Table 2).

Table 3 shows the distribution of scores according to the risk of disease per factor.

**Table 3:** Distribution of scores according to the risk of disease per factor, Uberlândia, MG, Brazil, 2019

Factors	Risk classification n (%)			
	OD	Severe	Critical	Satisfactory
EACT				
Work organization		20 (10.7)	134 (71.6)	33 (17.6)
Working conditions		8 (4.3)	106 (56.6)	73 (39)
ECHT				
Physical costs		139 (74.3)	48 (25.7)	0 (0.0)
EADRT				
Physical harm	9 (4.8)	35 (18.7)	56 (29.9)	87 (46.5)

ECHT = Escala de Custo Humano no Trabalho; EACT = Escala de Avaliação do Contexto de Trabalho; EADRT = Escala de Avaliação dos Danos Relacionados ao Trabalho; OD = occupational diseases.

Table 3 shows each risk factor based on the sum of the serious and critical risk scores, and shows that 154 (72.3%) and 114 (60.9%) workers, respectively, are at risk of work-related diseases. Meanwhile, 33 (17.6%) and 73 (39%) workers were found in the satisfactory risk score, respectively, to be subject to work organization and conditions that should be encouraged and serve as a parameter for those in the other scores.

The physical cost factor of the ECHT had a mean of 3.9 (SD: 0.6), classified as a serious risk of disease, and 100% of the workers in this factor were found to be at risk of disease (combination of serious and critical) (Tables 2 and 3).

Table 3 shows that 100 (53.4%) workers may have work-related disease in the three working months prior to the survey, considering that a critical score means that work-related diseases are present. In this factor, the acceptable risk score show that 87 (46.5%) workers, despite being the best scored group, have already suffered or may suffer some physical harm and deserve the same attention as all other workers.

## DISCUSSION

The results obtained allow us to analyze and discuss, in detail, the indications of risks of occupational disease. The factors and items that showed a serious risk indicate a strong risk of occupational disease, thus the need for immediate action to prevent it. Critical risk indicates that occupational disease is at a critical level, requiring attention and action in the short and medium term. When risks are classified as satisfactory, this indicates that workers are not getting sick as a result of the factor or item, which should be strengthened in the workplace. For the “physical harm” factor, the analysis is different, since it already includes situations of occupational pain and disorders. From critical risk, disease is found, and a score below critical is classified as acceptable.^12^

The authors of the ITRA suggest that one of the ways of interpreting the mean values is based on segmented exposure to each risk because even if the values for occupational disease risk are low, they are still a cause for concern and workers should have their health treated and cared for.^12^

A similar study including a group of workers from an outsourced janitorial and maintenance contractor at a federal public education institution resulted in mean scores of critical for the work organization factor and satisfactory for the working conditions factor and summed 54.54% and 30% of serious and critical risks for both factors, respectively.^15^

Another similar survey including a group of outsourced workers in various support services in a commercial building showed mean critical scores for work organization factor and satisfactory for working conditions factor and summed 66.67% and 36.67% of serious and critical risks for both factors, respectively.^16^

Based on the results of similar studies, it is clear that the work organization factor is constantly assessed as a critical risk of disease in outsourcing contractors. This study differs from the above-mentioned studies on the working conditions factor because while they all point to satisfactory assessment, this one presents this factor as critical risk of disease. However, Reis^15^ found that this positive result may have been biased due to the participants’ fear of reprisals from their employer.

The factors of work organization and working conditions have often been pointed out as leading to and/or causing accidents and diseases, disconnected from the fragile and limited focus on environmental risks known in OSH standards (physical, chemical, biological, ergonomic, and mechanical), human error, and analysis of the place where the accident occurred.^17,18,19,20,21^

Llory & Montmayeul^20^ describe the combination of factors that exceed what is prescribed in the rules as macro-determinants. These involve an in-depth safety analysis that includes the organization and working conditions, and other factors, identifying a set of problems that when addressed immediately have very effective preventive effects, eliminating the practice of blaming the workers involved in an accident.

Oliveira^21^ set forth a model for risk analysis, pointing out that accidents are linked to multifactorial causes, and that these factors should be investigated to identify a number of factors in advance, including work organization and environmental (conditions), and behavioral aspects, which together contribute to occupational accidents and diseases.

It is therefore clear that both factors of the EACT scale have become an important variable for OSH management and/or to understand the underlying causes of occupational accidents or diseases. Considering that more than half of the group surveyed is at critical risk of becoming ill from both factors, the contractor and the contractee need to jointly adjust the contractually agreed work to the actual conditions of the work environment as soon as possible. This should be done with the mutual participation of workers in their workplaces, so that the contractor and the contractee stay balanced and aligned with each other on OSH issues, so as to reduce the scores and reach a satisfactory level.^12,16^

The physical cost factor consists of the physical and motor demands on workers. A study with a group of janitorial and cleaning workers at a public hospital found that 87% of the participants had work-related musculoskeletal disorders, showing the remarkable vulnerability of workers in this sector to develop some kind of biomechanical pathology.^22^

Paula & Moraes^16^ found that the group surveyed was classified as being at critical risk of becoming ill as a result of the physical cost, with 56.57% for work organization. Reis,^15^ on the other hand, found that this factor resulted in the same descriptive values as our study, reaching the serious risk of disease, and with 100% of workers between critical and serious scores.

The literature recommends the most effective solution for controlling and reducing physical costs is the implementation of Ergonomic Workplace Analysis, which enables the inconsistencies between prescribed work and actual work to be contrasted. Thus, critical and vulnerable points can be identified, inadequate task arrangements, and more, so that specific solutions can be devised to impact on organizational factors and working conditions. Furthermore, these solutions, which provide for the reorganization of work processes and environment, have a positive impact on the OSH conditions of workers, their productivity, and the company as a whole.^23,24,25^

We believe that to reduce the scores of the three factors already discussed to satisfactory levels, systematic changes in work procedures and management are needed, and more efficient instruments and tools can be introduced with technical recommendations from OSH professionals and with the effective participation of workers, who are the main individuals performing the activities. It is important that whenever changes are made to any factor, they are duly monitored to verify their effectiveness, and to check for the appearance of new hidden or unseen causes of harm, a very common situation, highlighted Dejours.^18^

Pain and somatic disorders are the end products that emerge from the critical and serious results of the above factors. Unfortunately, it is common practice in Brazilian companies to restrict their actions to treating the worker’s symptoms or, even worse, to dismiss the worker and replace them with another one who is healthy, demonstrating no interest in seeking out the source of the problem to resolve it, perpetuating not only the physical effect, but the entire chain of factors that feed it and are the real causes of their suffering.^26^

A study by Paula & Moraes^16^ found a moderately high risk of illness for workers due to physical (53.33%), social (75.00%), and psychological (71.67%) factors. The remaining percentages within each category were distributed among “critical,” “serious,” and “presence of occupational diseases,” respectively. This classification represents the most positive outcome within this factor. These studies indicated a cumulative risk of 46.67% for physical damage, 25% for social damage, and 28.34% for psychological damage among workers with signs of illness risk.

Occupational pain and disorders can be seen as occupational accidents, according to a legal definition provided in article 19 of Law No. 8,213 of July 24, 1991^27^ because they cause functional disorders. Meanwhile, ITRA showed that a considerable number of times various injuries occurred within a 3-month period and no Comunicação de Acidente de Trabalho (CAT, Work Accident Report) was issued, indicating underreporting as found in the literature.^8,28^

It was also found that no health alterations were recorded in the annual report of the Programa de Controle Médico de Saúde Ocupacional (PCMSO, Occupational Health Medical Control Program). This means that diseases are not detected through anamnesis and clinical/complementary examinations conducted by occupational physicians, resulting in a loss of function to preserve and promote occupational health and an inability to detect symptoms of occupational pain and disorders. These inadequacies mean that ill employees continue to work with no medical support.^26,28,29^

It is therefore intended that, to mitigate physical harm, the company and their OSH team should delve deeper into detecting occupational symptoms, making diagnoses and, consequently, looking for the causes of occupational pain and disorders, so as to enable and take action to eliminate, neutralize or, at the very least, reduce the risk of occupational disease.^26^

This study has limitations including the fear of the participants of having their individual results identified and presented to senior managers (this may have biased the answers to certain items); the limited literature on the same population of the study with a view to OSH to better endorse the results and discussions; and the predominant use of the ITRA for studies on occupational pleasure and mental suffering, addressing OSH issues in a superficial and parallel manner.

## CONCLUSIONS

The diagnoses obtained on the risks of work-related diseases associated with specific factors related to occupational health call for urgent interventions, since strong indications of diseases, and possible signs and symptoms (pain and disorders) of diseases were detected among outsourced workers. However, it is worth saying that the diagnoses found through ITRA only have the capacity to make the risk of disease visible, and do not allow for the precise identification of the causes behind its onset. This requires further academic studies based on surveys and observations to find the possible causes, not excluding technical and professional actions to contribute to the same objective.

Although the results already show critical and serious scores for the risks of disease, these results could have been further intensified, since workers were completing the questionnaires, it was seen that the participants were afraid to write down certain answers for fear that senior managers would have access to the individual results and that they would suffer reprisals, even though all the ethical clarification of the research was given and they were fully aware of the confidentiality of the data and the beneficial purpose of the research.
